# Quality and timeliness of emergency obstetric care and its association with maternal outcome in Keren Hospital, Eritrea

**DOI:** 10.1038/s41598-022-18685-9

**Published:** 2022-08-26

**Authors:** Henos Kiflom Zewde

**Affiliations:** Department of Family and Community Health, Ministry of Health Anseba Province, Keren, Anseba Eritrea

**Keywords:** Health care, Medical research

## Abstract

Despite the critical role quality comprehensive emergency obstetric care (CEmOC) plays in ensuring safe motherhood, only a few studies have attempted to measure the impact of substandard and delayed care on maternal outcome thus far. This study evaluates the association between various process and timeliness indicators of CEmOC and adverse maternal outcome in Keren Hospital. This study compared women with potentially life-threatening condition (PLTC) and women with severe maternal outcome (SMO) with respect to various process and timeliness indicators. Logistic regression analysis was employed to assess the association of timeliness and process indicators with SMO using SPSS version-22 computer software. In this study, we included 491 cases of PLTC and 210 cases of SMO (171 maternal near misses and 39 maternal deaths). The following process indicators showed significant association with SMO: failure to give uterotonics for the treatment of postpartum hemorrhage, failure to administer prophylactic antibiotics, and delayed laporatomy for uterine rupture. Moreover, delays in referral, triaging, seeing an obstetrician, and receiving definitive treatement were strongly associated with SMO. The following causes of delay were also found to be independently associated with SMO: erroneous diagnosis, inappropriate management, multiple referrals between health facilities, unavailability of a senior obstetrician, and poor communication during referral. Among the miscellaneous factors, nighttime admission and referral during the rainy season showed significant association with SMO. Findings of this study indicate that huge gap exists in providing quality and timely care in Keren Hospital. In general, most incidents of substandard and delayed care were due to poor referral system, insufficiency of medical staff, inadequacy of drugs and equipment, and unavailability of standard management protocol. Improving the referral system, upgrading the technical skills of health professionals, making sure life-saving drugs and equipment are available all the time, and posting standard treatment and management protocols in the maternity and emergency rooms will play a vital role in reducing the occurrence of SMO in Keren Hospital.

## Introduction

Currently, the international community is striving to reduce the rate of maternal mortality to less than 70 per 100,000 live births by the end of this decade^1^. To achieve this goal, most developing countries have put strategies of essential obstetric care (EOC), including quality antenatal care and skilled birth attendance, at the heart of their maternal health agenda^2^. However, such strategies are futile without quality emergency obstetric care services in place to manage maternal complications adequately^2^. Most maternal complications are difficult to predict and prevent since any pregnant woman can develop them at any time during pregnancy, delivery, or in the postpartum period. Thus, provision of effective, affordable and quality emergency obstetric care (EmOC) and efficient referral system plays pivotal role in the endeavor of promoting safe motherhood.

Due to the low institutional delivery coverage in most developing countries^3^, a large proportion of women reach health facilities after they develop serious maternal complications. Hence, provision of accessible and quality emergency obstetric care services is of profound importance in such countries^4^. There are a number of recommended process and timeliness indicators useful for the evaluation of the quality of emergency obstetric care. Process indicators are used to evaluate the presence of a set of emergency procedures (or lack of them) to deal with maternal complications^5^. Timeliness indicators, on the other hand, are useful to measure if the required procedures are being done without significant delay^6^.

Several studies from developing countries previously utilized the WHO maternal near miss (MNM) tool to expose deficiencies in the quality of life-saving medical interventions in comprehensive emergency obstetric care (CEmOC) facilities^7–10^. In a study conducted in Tanzania^8^, for instance, of all women who delivered in the hospital, only 48% received uterotonics beforehand. The study further revealed that only 42% of women with postpartum hemorrhage received uterotonics, and only 66% of those who underwent caesarean section took prophylactic antibiotics. Another study from Brazil reported drawbacks in the timeliness of key obstetric interventions^11^. According to the study, delay in diagnosis, inadequate skills of medical professionals, and improper management were the main factors that undermined the quality of care.

While the fact that delayed and poor-quality emergency obstetric care is prevalent among women who experience adverse maternal outcome is well-established, only a few studies attempted to compare women with adverse maternal outcome to those with favorable outcome, and thereby to measure the magnitude of association between maternal outcome and quality of care. To measure such association, a study from Indonesia^12^ used a case–control study, where maternal deaths were considered as cases and mothers with organ dysfunction as controls. However, this method is not practically sound in most resource-poor facilities, including ours, for two obvious reasons. First, maternal deaths are very few in absolute number in most facilities. Hence, the power of the study can be seriously compromised due to inadequate sample size. Second, organ dysfunction is a near-death condition on its own right, which should be prevented from occurring^13^. Therefore, the same pitfalls in quality of care, which are responsible for maternal deaths, are more likely to occur in most cases of organ dysfunction as well.

In this study, we compared women who merely experienced potentially life threatening-condition (PLTC) and those with severe maternal outcome (SMO)^5^ with respect to a set of process and timeliness indictors of EmOC. These two maternal groups are similar in every aspect except for the ultimate outcome of interest^5^. Hence, it is expected that comparing them with regard to factors related to quality and timeliness of care would shed light on the magnitude of the role such factors play in determining maternal outcome. The main objectives of this study were, therefore, to assess the quality and timeliness of emergency obstetric care in Keren provincial referral hospital and to measure its association with maternal outcome.

## Methods

### Study setting

This study was conducted in Anseba province, Eritrea. Anseba province is one of the six provinces of Eritrea. Keren Hospital is the only referral facility providing comprehensive emergency and obstetric care (CEmOC) for women with maternal complications in the entire province. It is a public hospital that serves women referred by community hospitals, health centers, and health stations in surrounding districts. In total, the hospital accommodates 80 beds in the maternity ward. It provides reproductive health, child health, and CEmOC service free of charge. On average, the hospital attends around 5000 deliveries annually. It provides service for the estimated 549,000 residents of the province. All pregnant women who were in labor, delivered or aborted, or within 42 days of the postpartum period, and admitted to the maternity or emergency wards of this hospital from April 2018 to April 2021 were the source population for this study.

### Study design

A facility-based unmatched case–control study was conducted to address the objectives of the present study.

### Inclusion and exclusion criteria

As recommended by the WHO MNM approach^5^, identification of near-miss mothers followed a two-step process. In the first step, women who were pregnant, in labor, aborted, or within 42 days of termination of pregnancy who met the criteria of “potentially life-threatening condition” according to the WHO MNM approach were selected. Then, all women in the PLTC category who fulfilled the WHO life-threatening conditions (near-miss criteria) were included as cases in this study, while the remaining mothers who failed to meet the WHO maternal near-miss criteria were enrolled as controls. Women whose medical records were unavailable at the time of data collection or contained a considerable amount of missing data were excluded from the study.

### Study variables

#### Dependent variable

Maternal outcome was the variable designated as a dependent variable in this study. For the purpose of this study, all women with PLTC were categorized in two groups. Women with SMO based on the WHO MNM criteria were classified as cases while those with only PLTC were considered as controls. Operational definition of the dependent variables included in this study can be found as supplementary material (Supplementary file).

#### Independent variables

These are variables related to quality of obstetric care, which are included in this study as predictors of maternal outcome. All independent variables are classified in one of the following categories: variables related to operational quality of care, variables related to timeliness of care, and miscellaneous variables. Operational definition of the independent variables included in this study can be accessed from the supplementary material (Supplementary file).

#### Variables related to operational quality of care

All process indicators proposed in the WHO MNM approach^5^ were used as a proxy to measure the operational quality of care in Keren Hospital.

#### Variables related to timeliness of care

In this study, the framework proposed by Edson et al.^6^ was used, with some modification, to assess the timeliness of obstetric care. Intervals between critical events were evaluated to identify intervention-specific delays. The critical events considered for this purpose were decision to make referral, arrival at hospital, making triage, seeing a senior obstetrician, making diagnosis, and administering definitive treatment. Moreover, the following causes of delay were also treated as independent variables in this category: poor communication during referral, senior staff unavailable, erroneous diagnosis, lack of supplies and equipment, inappropriate management, and patient related delay. Since there is no standard definition of delay, the decision whether significant delay occurred was entirely based on the subjective judgment of the reviewers.

#### Miscellaneous variables

Variables outside the direct control of the health system that can have substantial impact on the quality of emergency obstetric care were also included as independent variables in this study. Referral during the rainy season, nighttime admission, and weekend admission were the variables included in this category.

### Sample size determination

The sample size for the present study was estimated using Epi info-7 software employing the formula of sample size determination for unmatched case–control studies. The following assumptions were made while calculating the sample size: confidence level of 97%, power of 80%, case to control ratio of 1:2, proportion of controls exposed 52%, and proportion of cases exposed 70.8%. The proportion of exposure among cases and controls was taken from a Brazilian study^11^. The percentage of “third delay” documented in the Brazilian study was the exposure variable considered when calculating the proportion of exposure among cases and controls. The calculation yielded a minimum sample size of 100 cases and 200 controls. After adding 10% for data unavailability, the final sample size was calculated to be 110 cases and 220 controls. However, we opted to include all cases of PLTC and SMO identified during the entire study period to increase the power of the study.

### Data collection

Data for the present study was collected by two senior obstetricians who provide obstetric care in Keren Hospital. They were trained on how to identify women who experienced potentially life-threatening conditions during pregnancy, delivery, or the postpartum period based on the WHO eligibility criteria^5^. Once they reviewed the medical records thoroughly, they selected all eligible women from the registries of antenatal care ward, delivery ward, obstetric ward, and emergency ward. After identifying all eligible women, the data collectors selected all women fulfilling the MNM criteria using the WHO MNM checklist. All maternal deaths were also extracted from the medical registers and were classified in the SMO group along with the WHO MNM cases.

### Data analysis

Once collected, the data was entered and cleaned using Epi Info 7 software, and then analyzed using SPSS version-22 computer software. Descriptive statistics were used to summarize the baseline characteristics of study participants. Differences between cases and controls with regard to demographic, obstetric, and medical characteristics were assessed using Pearson’s chi square test (χ^2^). A dummy table prepared by the WHO technical working group^5^ was used to indicate process indicators related to specific conditions. Binary logistic regression analysis was then used to assess which process indicators were associated with SMO. Timeliness in the provision of emergency obstetric care and its association with SMO was analyzed based on the framework proposed by Edson et al.^6^, with some modifications. Multivariate logistic regression analysis was also performed to evaluate the association of delays and their causes with SMO. In addition, miscellaneous variables that may affect the association between quality of care and maternal outcome were considered in the analysis. Crude or adjusted odds ratio alongside the 95% confidence interval was provided whenever logistic regression analysis was employed. The p-value for the Pearson’s chi square test and logistic regression analysis was set at 0.05.

### Data quality assurance

Data for the current study was collected by two competent and senior obstetricians who have been providing CEmOC service for more than five years in different referral hospitals. They were given training on how to identify relevant registers, how to extract information from them, and how to handle unclear and missing data. Clear and detailed explanation of the WHO MNM identification process was given to all data collectors to minimize inter-observer and intra-observer discrepancies. Data collectors were strictly informed to contact the principal researcher in case of any ambiguity or uncertainty. Such ambiguities were then resolved based on consensus between the researcher and the data collectors. The principal researcher closely monitored the entire data collection process, and registers were revisited in case of any doubt.

### Ethical consideration

The entire study process was strictly abided by acceptable ethical standards. Approval was sought from provincial and national health research review and ethics committees. Adequate explanation was given to all concerned bodies regarding the purpose and benefits of the study. The study was conducted in accordance with the guidelines for research outlined by the national research and ethics committee and the Helsinki declaration of 1975, as revised in 1983. Written consent to review medical records was obtained from the medical director of Keren Hospital. The need to obtain informed consent was waived by the national research and ethics committee as this is the standard procedure for research activities entirely based on routinely collected data. In order to revise records in case of doubt and to avoid repetition of cases recorded in multiple registers in different wards essential participants’ identifiers were recorded in a logbook. The logbook was kept in a secure cabinet until data collection period was over, and all recorded information was destroyed soon after the completion of the study. Likewise, all data obtained using the data abstraction tool were de-identified following completion of the study, so that none of the collected information could be tracked back to any individual participant.

## Results

In this study, we reviewed medical records of 210 SMO cases and 491 controls. Of the 210 cases of SMO, 171 were MNMs and the remaining 39 were maternal deaths. The majority of study participants were between the ages of 20 to 34 and parity of one to three. Significantly higher proportion of mothers in the SMO group were urban residents compared to controls. In both groups, majority of the mothers delivered at term and did not have any preexisting chronic medical condition. More women in the SMO group underwent caesarean section compared to controls, and this difference was statistically significant (Table 1).

**Table 1:** Demographic, obstetric and medical characteristics of women with potentially life-threatening conditions admitted to Keren Hospital.

Characteristics	SMOs	Controls
	N (%)	N (%)
Age in years*		
≤ 19	62 (29.5)	110 (22.4)
20 to 34	99 (47.1)	218 (44.4)
≥ 35	49 (23.3)	163 (33.2)
Parity*		
Null	54 (25.7)	103 (21)
1 to 3	95 (45.2)	183 (37.3)
4 and above	61 (29)	205 (41.8)
**Gestational age**		
< 36 weeks	27 (12.9)	54 (11)
≥ 36 weeks	183 (87.1)	437 (89)
Residence**		
Urban	168 (80)	291 (59.3)
Rural	42 (20)	200 (40.7)
Mode of delivery**		
Spontaneous delivery	24 (11.4)	164 (33.4)
Caesarean section	186 (88.6)	327 (66.6)
**Pre-existing medical conditions**		
Yes	16 (7.6%)	47 (9.6%)
No	194 (92.4%)	444 (90.4%)

Chi-square test **significant at p = 0.001, *significant at p = 0.05.

*SMO* severe maternal outcome.

About one third (205) of all women eligible for this study were diagnosed with severe post-partum hemorrhage. Of those, 89 (78.8) from the control group and 59 (64.1%) from the SMO received uterotonics as a treatment. Similarly, we documented 163 (23.3%) cases of eclampsia, almost all (98.1%) of whom received anticonvulsants. Approximately half (50.8%) of the women included in this study underwent caesarean section. Prior to the procedure, only 69% of those from SMO group and 82.9% from the control group received prophylactic antibiotics. There were 120 (17.1%) mothers who developed severe systemic infection, and 89.2% of them were treated with parenteral antibiotics. Uterine rupture was documented as the principal diagnosis in the medical records of 102 (14.6%) women. Only 34.2% of cases and 59.4% of control women with ruptured uterus underwent laparotomy within 3 h of hospital stay. Corticosteroids were administered to 49.3% of women who delivered preterm. The highest case fatality rate recorded was for sepsis (12.5%), while uterine rupture had the lowest case fatality rate (6.9%) (Table 2).

**Table 2:** Process indicators of specific obstetric conditions among women admitted to Keren Hospital.

Indicators	SMOs	Controls
	N (%)	N (%)
**Treatment of severe postpartum hemorrhage**		
Women with severe PPH	92 (43.8)	113 (23)
Any uterotonic given	59 (64.1)	89 (78.8)
Mortality	9.3%	
**Anticonvulsants for eclampsia**		
Women with eclampsia	60 (28.6)	103(21)
Any anticonvulsant given	58 (96.7)	102 (99)
Mortality	9.2%	
**Prevention of caesarean section related sepsis**		
Women undergoing caesarean section	145 (69)	211 (43)
Prophylactic antibiotic during caesarean section	100 (69)	175 (82.9)
**Treatment of sepsis**		
Women with sepsis	46 (21.9)	74 (15.1)
Parenteral therapeutic antibiotic	43 (93.5)	64 (86.5)
Mortality	12.5%	
**Management of ruptured uterus**		
Women with ruptured uterus	38 (18.1)	64 (13)
Laparotomy after 3 h of hospital stay	25 (65.8)	26 (40.6)
Mortality	6.9%	
**Corticosteroids for lung maturation in preterm birth**		
Women having preterm delivery after 3 h of hospital stay	27 (3.9)	54 (7.7)
Corticosteroids for fetal lung maturation	14 (51.9)	26 (48.1)

When binary logistic regression analysis was employed, the following process indicators showed statistically significant association with SMO: uterotonics for the treatment of PPH (OR: 2.07; CI: 1.12–3.86), prophylactic antibiotics before caesarean section (OR: 2.19; CI: 1.32–3.62), and laparotomy within 3 h of hospital stay for uterine rupture (OR: 2.81; CI: 1.22–6.48) (Table 3).

**Table 3:** Bivariate logistic regression analysis of process indicators as determinants of maternal outcome among women admitted to Keren Hospital.

Indicators	COR	95% CI
Uterotonic for the treatment of PPH	2.07*	1.12–3.86
Anticonvulsants for the treatment of eclampsia	N/A	N/A
Prophylactic antibiotics	2.19*	1.32–3.62
Therapeutic antibiotics	N/A	N/A
Laparotomy within 3 h of hospital stay	2.81*	1.22–6.48
Corticosteroids for fetal lung maturation	0.86	0.34–2.17

*Significant at p = 0.05, N/A-not applicable since there are cells containing less than 5 observations.

Delay in referral was the most frequently documented type of third delay in both groups. Delay in making diagnosis on the other hand, was the least frequently recorded delay among the SMO group as delay in triage was among the control group. Over two thirds (69%) women in this study experienced at least one type of third delay. Compared to the control group, women with SMO were more likely to experience delay in referral (OR: 2.23; CI: 1.53–3.23), triage (OR: 4.01; CI: 2.45–6.62), seeing an obstetrician (OR: 2.86; CI: 1.86–4.39), receiving a definitive treatment (OR: 4.93; CI: 3.26–7.47), and any type of the third delays (OR: 3.22; CI: 2.13–4.86) (Table 4).

**Table 4:** Timeliness indicators as determinants of maternal outcome among women admitted to Keren Hospital.

Third delay	SMO (%)	Controls (%)	AOR	CI (95%)
Delay in referral	95 (52.8)	157 (38.1)	2.23**	1.53–3.23
Delay in triage	50 (23.8)	39 (7.9)	4.03**	2.45–6.62
Delay in seeing obstetrician	67 (31.9)	63 (12.8)	2.86**	1.86–4.39
Delay in making diagnosis	40 (19)	112 (22.8)	0.71	0.45–1.11
Delay in definitive treatment	86 (41)	59 (12)	4.93**	3.26–7.47
Any of the above delays	177 (84.3)	307 (62.5)	3.22**	2.13–4.86

*Significant at p = 0.05.

Unavailability of a senior obstetrician at the time of admission was the main cause of third delay in both categories of maternal outcome, while patient related delay was the least reported cause of delay. In the final multivariate logistic regression analysis, the following causes of delay were found to be independently associated with SMO: lack of supplies and equipment (OR: 2.98; CI: 1.61–5.52), inappropriate management (OR: 3.8; CI: 2.31–6.25), multiple referrals between health facilities (OR: 4.46; CI: 2.16–9.2), unavailability of a senior obstetrician (OR: 1.57; CI: 1.05–2.36), and poor communication during referral (OR: 2.59; CI: 1.61–4.17) (Table 5).

**Table 5:** Major causes of delay and their association with maternal outcome among women admitted to Keren Hospital.

Causes of delay	SMO (%)	Controls (%)	AOR	CI (95%)
Erroneous diagnosis	28 (13.3)	29 (5.9)	1.43	0.75–2.7
Lack of supplies and equipment	41 (19.5)	22 (4.5)	2.98**	1.61–5.52
Inappropriate management	66 (31.4)	40 (8.1)	3.8**	2.31–6.25
Multiple referrals	22 (10.5)	15 (3.1)	4.46**	2.16–9.2
Senior obstetrician unavailable	115 (54.8)	141 (28.7)	1.57*	1.05–2.36
Poor communication in referral	61 (29)	56 (11.4)	2.59**	1.61–4.17
Patient related delay	8 (3.8)	22 (4.5)	0.95	0.39–2.32

*Significant at p = 0.05.

Women with SMO were more likely to be admitted during the nighttime (OR: 2.9; CI: 2.03–4.14). Similarly, women referred during the rainy season had almost four times (OR: 3.82; CI: 2.63–5.56) higher odds of experiencing SMO incident. We were not able to establish significant association, however, between weekend admission and SMO (Table 6).

**Table 6:** Miscellaneous variables related to quality of obstetric care as determinants of maternal outcome among women admitted to Keren Hospital.

Variables	SMO (%)	Controls (%)	AOR	CI (95%)
Night admission	104 (49.5)	117 (23.8)	2.9**	2.03–4.14
Weekend admission	33 (15.7)	89 (18.1)	0.86	0.54–1.38
Admitted during rainy season	93 (44.3)	79 (16.1)	3.82**	2.63–5.56
Preexisting medical conditions	16 (7.6)	47 (9.6)	0.78	0.42–1.48

**Significant at 0.01.

## Discussion

In the present study, we compared the quality of maternal care in women who merely developed PLTC and those who developed SMO. The results suggest that improvement in the quality and timeliness of key obstetric interventions can significantly reduce the occurrence of undesirable maternal outcome.

Findings of this study revealed that deficiencies in the referral system were among the strongest predictors of SMO (OR: 2.23; CI: 1.53–3.23). Some previous studies regarded all referral related delays as delays in reaching health facilities from home^14,15^. However, referral delays are not necessarily type II delays. Certain referral delays, especially those that occur after the mother reaches a lower level facility, are mostly caused as a result of health system failures. At times, staff members at lower level facilities fail to recognize life-threatening conditions early^16^. Moreover, once the decision to refer the mother is made, difficulties may arise in securing an ambulance for transport^16–18^. Anseba province has eleven ambulances, most of which were in poor condition during the study period. All districts, except one, have only one functional ambulance to cover their entire catchment area. All ambulances are state owned and are rationed 200 L of benzene per month. Should any health facility encounter an emergency obstetric referral while ambulance fuel is out of stock, family members of the mother are compelled to provide the required amount of fuel. Thus, loopholes in ambulance service may explain a substantial portion of the excess adverse outcomes attributable to referral delays.

This study revealed another dimension of the referral system failure as well: delays due to multiple referrals (OR: 4.46; CI: 2.16–9.2). In Eritrea, there is no any written referral protocol intended for obstetric emergencies. However, all facilities are informed to refer any emergency, including obstetric emergencies, directly to the next higher organizational unit nearest to them. Health stations refer to health centers, health centers to provincial referral hospitals, and provincial referral hospitals to national referral hospital. Since health stations are on the frontline of all emergencies, they are most likely to be the first to encounter women in dire obstetric conditions. Most health stations follow the recommended referral chain and, therefore, refer the mothers to their respective district health centers. However, none of the district health centers in Anseba province has the capacity to perform beyond the basic signal functions. As a result, majority of referred mothers, especially those with severe PPH, obstructed labor, and ruptured uterus, are referred again to the provincial referral hospital, having wasted valuable time in the health centers. Several similar studies also documented delay due to multiple referrals^18–20^. Poor communication between referring and receiving facilities was another cause of delay that showed significant association with SMO in the present study (OR: 2.59; CI: 1.61–4.17). As is the case in most previous studies^11,16,21,22^, almost all health centers and health stations in Anseba province often fail to notify the recipient facility in advance when referring women with obstetric emergencies. Furthermore, referring patients with an incomplete referral form^22^ and failure of recipient facilities to provide feedback to referring facilities^21,22^ may be additional factors that compromise effective communication during referral in Keren Hospital.

Huge deficits in the referral system means that a number of women will inevitably reach CEmOC facilities in desperate condition having experienced significant delays. Hence, in such facilities, putting an effective and responsive triage system in place is of paramount importance^23^. Keren Hospital does not have any formal triaging protocol. Staff members categorize obstetric patients arbitrarily based on their subjective judgments. Moreover, we found that approximately one fourth of all women with SMO waited for more than 30 min before they were evaluated. Previously, incompetency of medical professionals, shortage of technical staff and lack of diagnostic equipment were identified as the main factors leading to delays in obstetric triage^24,25^. Goodman et al.^26^ reported that improving the triage room, using colored wristbands, and applying a triage assessment form can significantly improve the quality and timeliness of obstetric triages.

Unfortunately, timely and effective triage system cannot secure a favorable maternal outcome on its own. Once triaged, patients may experience further delays in seeing a senior obstetrician^18,27^. In the present study, women who experienced delay in seeing a senior obstetrician were almost three fold (OR: 2.86; CI: 1.86–4.39) more likely to encounter adverse maternal outcome. There are only two obstetricians competent enough to perform emergency obstetric surgical operations in Keren Hospital. Hence, a significant proportion of deliveries occur “out-of-hours”, and an ambulance has to pick either of them from home whenever an emergency case arrives while both of them are off duty.

This study showed that the overall score of process indicators for both categories of women was below par. Even though provision of prophylactic antibiotics is the recommended protocol to prevent post-caesarean section systemic infection^28^, only 77.2% of those who underwent caesarean section received prophylactic antibiotics. There was substandard care in the treatment of ruptured uterus as well. Half of all women with a ruptured uterus waited for more than 3 h before laparotomy was performed, despite WHO’s recommendation to perform laparotomy within three hours of hospital stay^5^. In line with our findings, studies done in various developing countries reported several accounts of substandard care among women who experienced SMO^7–10^. Our study is, however, the first one to provide objective estimates on the degree of association between substandard emergency obstetric care and poor maternal outcome. Compared to the “merely potentially life-threatening group”, women in the SMO group had higher odds of receiving substandard treatment of PPH (OR: 2.07; CI: 1.12–3.86), not to take prophylactic antibiotic (OR: 2.19; CI: 1.32–3.62), and to undertake a delayed laparotomy for ruptured uterus (OR: 2.81; CI: 1.22–6.48).

Adverse maternal outcomes occur not only due to substandard obstetric care though, but also due to delays in providing the required medical interventions. This study showed that in all critical events considered for this study, except for diagnosis, delays were more likely to occur in women who experienced SMO. Of all types of third delay, delay in providing definitive treatment was the one that showed the strongest association with adverse maternal outcome (OR: 4.93; CI: 3.26–7.47). This finding was further corroborated by the fact that lack of medical supplies (OR: 2.98; CI: 1.61–5.52) and inappropriate management (OR: 3.8; CI: 2.31–6.25), both of which impede timely and effective treatment, were among the most important predictors of unfavorable maternal outcome. Since the blood bank in our hospital is inadequately stocked and poorly equipped, the majority of delays in definitive treatment were because of blood transfusion related delays. Various studies reported similar findings as well, where lack of essential life-saving equipment and supplies (including blood for transfusion) caused significant delays^27,29–33^. Incorrect management was another important reason for delayed definitive treatment. Several factors are believed to contribute to this finding, among which are unavailability of written standard treatment protocols in the delivery and emergency rooms^27,29^, inadequate staff competency^11,17,33^, and poor communication among technical staff^11^-especially communication with laboratory personnel.

Factors that are potentially related to both quality of care and maternal outcome that could easily confound the observed association were handled with special care in the present study. The effect of seasonal variation in the proportion of SMO cases in Keren Hospital was the most important of these factors. Four of the nine districts in Anseba province have to cross Anseba River to reach the provincial referral hospital in Keren. Anseba River, one of the largest and longest seasonal rivers in Eritrea, blocks transportation to and from these districts during the rainy season. It is capable of restricting movement for hours and sometimes-even days, depending on the amount and duration of rainfall in the highlands. Hence, we hypothesized that more women during the rainy season would encounter referral delays and thus develop adverse maternal outcome. Concordant with our hypothesis, women with SMO were found to be four (OR: 3.82; CI: 2.63–5.56) times more likely to be admitted during the rainy season. Nevertheless, the association between maternal outcome and referral delay remained significantly strong even after controlling the effect of seasonality on maternal outcome. Makanga et al.^34^ have discussed in detail how seasonality affects access to emergency obstetric care employing a spatial–temporal study design.

Our study revealed that there was a three-fold (OR: 2.9; CI: 2.03–4.14) increase in the risk of adverse maternal outcome for women admitted during the nighttime compared to those admitted during the daytime. In line with our findings, studies conducted in Mexico and Ghana^35,36^ reported a significantly higher maternal mortality rate in the night shift. Shortage of staff and lack of certain drugs and equipment during the night shift may explain the increased likelihood of adverse maternal outcome during the nighttime.

There is mixed evidence in the available literature regarding the “weekend effect” in relation to maternal outcome, with some studies reporting a positive association between maternal outcome and weekend admission^37^ while others fail to establish a similar association^38,39^. Weekend admission did not show any significant association with maternal outcome in this study. This result was concordant with the findings of a study from the UK^39^. The fact that at least one senior obstetrician is obliged to attend during the weekends, as opposed to the nighttime when obstetricians are off-duty, in Keren Hospital may explain the absence of an association between maternal outcome and weekend admission. The study from the UK^39^, however, failed to document any effect of weekend admission on maternal outcome even in the absence of senior consultant during the weekends.

### Strengths and limitations

This study is one of the few studies that measured the impact of process and timeliness indicators on maternal outcome using analytical study design. Our study, however, differs from previously conducted similar studies since it compared women with PLTC and SMO: maternal groups with similar prospect of encountering adverse maternal outcome, yet one group experiencing satisfactory outcome while the other ends up with SMO. We were able not only to reveal various factors associated with quality and timeliness of care, but also to prioritize them based on the strength of their association. This study evaluated the effect of several causes of delay on maternal outcome as well. Similarly, major confounding factors associated with both quality of care and maternal outcome were also taken care of in this study to make an unbiased estimate.

The study has its own limitations though, the most notable one being the fact that it was based on secondary data. Data gathered from medical registers is heavily dependent on the quality of the hospital’s data handling system. Poor quality data due to mistakes in registration, incomplete record keeping, and ambiguous handwriting are rampant in most poorly equipped and under-staffed facilities^40^. Fortunately, the quality of data obtained from Keren Hospital was deemed sufficient for the purpose of this study. Another drawback of this study is that it employed observational study design to achieve its objectives. Hence, it is not possible to establish a cause and effect relationship despite the strong association observed between various process and timeliness indicators and poor maternal outcome. The findings of this study, however, are expected to shed light on the effect of quality of care on maternal outcome in settings similar to ours.

## Conclusion

This study showed that an immense gap exists in providing quality and timely emergency obstetric care in Keren referral hospital. Failure to provide prophylactic antibiotics and waiting for more than 3 h to perform laparotomy for uterine rupture were the main weaknesses in quality of care. On the other hand, delays during referral, triage, seeing a senior obstetrician, and definitive treatment were the major factors that compromised the timeliness of care. In general, most incidents of substandard and delayed care were due to insufficiency of medical staff, inadequacy of drugs and equipment, and unavailability of standard management protocol. A number of limitations were also identified in the referral process, multiple referrals being the most important of them. Thus, efforts should be made to avoid delays in referral with special focus on the summer. Moreover, upgrading the skills and competencies of health professionals, making sure life-saving drugs and equipment are available all the time, and posting standard treatment and management protocols in the maternity and emergency rooms will play a vital role in reducing the occurrence of SMO in Keren Hospital.

## Supplementary Information

Supplementary Information.

## Data Availability

The complete data set supporting the conclusion of this article is available from the author and can be accessed upon reasonable request.

## Abbreviations

WHO: World health organization
CEmOC: Comprehensive emergency obstetric care
PLTC: Potentially life threatening conditions
SMO: Severe maternal outcome
MNM: Maternal near miss
SPSS: Statistical package for social sciences
OR: Odds ratio
CI: Confidence interval
