# Supplementary material for: Quality and timeliness of emergency obstetric care and its association with maternal outcome in Keren Hospital, Eritrea
Source: Sci Rep. 2022 Aug 26;12:14614. doi: 10.1038/s41598-022-18685-9 (PMC9418268; doi:10.1038/s41598-022-18685-9)
Supplement: Supplementary file 1 — Supplementary Information. [file 41598_2022_18685_MOESM1_ESM.pdf]

Quality and timeliness of emergency obstetric care and its association with maternal outcome in Keren hospital, Eritrea

Henos Kiflom Zewde [1]

Affliation

[1] Department of family and community health, Ministry of Health Anseba province, Anseba, Keren, Eritrea

Correspondence to: Henos Kiflom Zewde. E-mail: heniutd@gmail.com

Operational definition of dependent variables

Maternal near miss (MNM): refers to a woman who nearly died but survived a complication that occurred during pregnancy, childbirth or within 42 days of termination of pregnancy.

Maternal death (MD): is the death a woman while pregnant or within 42 days of termination of pregnancy or its management, but not from accidental or incidental causes.

Severe maternal outcome (SMO): refers to a life-threatening condition (i.e. organ dysfunction), including all maternal deaths and maternal near miss cases.

Potentially life-threatening conditions (PLTC): refers to five life threatening obstetric conditions i.e. severe post-partum hemorrhage, severe pre-eclampsia, eclampsia, sepsis (severe systemic infection), and uterine rupture.

Operational definition of independent variables associated with quality and timeliness of care

Operational definition of delays

Delay in referral: patient delay in arriving at the hospital after the decision for referral is made.

Delay in triage: delay in triaging the woman after arriving in the hospital.

Delay in seeing obstetrician: delay in being attended by an obstetrician having been given priority by the triage system.

Delay in making diagnosis: delay by the obstetrician attending the woman in making the correct diagnosis.

Delay in definitive treatment: delay in giving the woman the definitive treatment and/or management after making the right diagnosis.

N.B- the decision whether there is any delay in the above categories rests on the judgment of the obstetricians who collected data for this study.

Operational definition of causes of delay

Erroneous diagnosis: evidence of delay in making the correct diagnosis due to mistakes by health professionals.

Lack of supplies and equipment: evidence of delay in making triage, diagnosis, and/or treatment due to unavailability of certain medical supplies and equipment.

Inappropriate management: evidence of delay in making definitive treatment and management due to failure to comply with the standard treatment protocol.

Multiple referrals: evidence of delay in arriving at hospital following referral due to visits to one or more health facilities before making the journey to the hospital.

Senior obstetrician unavailable: evidence of delay in seeing obstetrician due to the absence of obstetrician in the hospital after the woman is triaged.

Poor communication in referral: evidence of delay in diagnosis and/or treatment because the referring facility failed to communicate effectively with the receiving hospital in advance during the time of referral.

Patient related delay: evidence of delay in making the diagnosis and/or treatment due to patient's disobedience and noncompliance.

N.B- the decision whether any of the above causes of delay contributed to the occurrence of third delay rests on the judgment of the obstetricians who collected data for this study.

Operational definition of miscellaneous independent variables

Night admission: admission of a woman between 6:00 pm and 8:00 am

Weekend admission: admission of a woman during the weekend (Saturday and Sunday)

Admission during rainy season: admission of a woman during the usual rainy season of Anseba province (i.e. from June 21 to September 21).

Preexisting medical conditions: the presence of any chronic comorbidity including diabetes, hypertension and other chronic heart diseases in a woman.
